# Potential Relationship between Phenotypic and Molecular Characteristics in Revealing Livestock-Associated *Staphylococcus aureus* in Chinese Humans without Occupational Livestock Contact

**DOI:** 10.3389/fmicb.2016.01517

**Published:** 2016-09-27

**Authors:** Yanping Fan, Xiaolin Wang, Ling Li, Zhenjiang Yao, Sidong Chen, Xiaohua Ye

**Affiliations:** School of Public Health, Guangdong Pharmaceutical UniversityGuangzhou, China

**Keywords:** community-acquired, livestock-associated, methicillin-resistant *S. aureus*, multidrug-resistant *S. aureus*

## Abstract

While some studies have defined *Staphylococcus aureus* based on its clonal complex and resistance pattern, few have explored the relations between the genetic lineages and antibiotic resistance patterns and immune evasion cluster (IEC) genes. Our aim was to investigate the potential relationship between phenotypic and molecular characteristics so as to reveal livestock-associated *S. aureus* in humans. The study participants were interviewed, and they provided two nasal swabs for *S. aureus* analysis. All *S. aureus* and methicillin-resistant *S. aureus* (MRSA) were tested for antibiotic susceptibility, multilocus sequence type and IEC genes. Of the 1162 participants, 9.3% carried *S. aureus*, including MRSA (1.4%) and multidrug-resistant *S. aureus* (MDRSA, 2.8%). The predominant multidrug-resistant pattern among MDRSA isolates was non-susceptibility to erythromycin, clindamycin and tetracycline. The most common *S. aureus* genotypes were ST7, ST6, ST188, and ST59, and the predominant MRSA genotype was ST7. Notably, the livestock-associated *S. aureus* isolates (IEC-negative CC9, IEC-negative tetracycline-resistant CC398, and IEC-negative tetracycline-resistant CC5) were found in people with no occupational livestock contact. These findings reveal a potential relationship between *S. aureus* CCs and IEC genes and antibiotic resistance patterns in defining livestock-associated *S. aureus* in humans and support growing concern about the potential livestock-to-human transmission of livestock-associated *S. aureus* by non-occupational livestock contact.

## Introduction

Methicillin-resistant *Staphylococcus aureus* (MRSA) is an important cause of nosocomial infections worldwide (Woodford and Livermore, 2009). The prevalence of MRSA in hospitals is approximately 50–80% in some Asian countries, such as Korea, Taiwan, Hong Kong, Thailand, and Vietnam (Song et al., 2011). Despite the high prevalence of healthcare-associated MRSA (HA-MRSA) in Asia, only a few epidemic clones have been identified; sequence type (ST) 239 has been reported in China, India, Thailand and Taiwan, and ST5 has been identified in Japan and South Korea (Ko et al., 2005). Recent reports have indicated that the epidemiology of MRSA is undergoing a major change following the emergence of community-associated MRSA (CA-MRSA) strains in patients without any risk factors or previous health-care contact (David and Daum, 2010; DeLeo et al., 2010). CA-MRSA outbreaks have been reported worldwide, and successful clones are usually associated with specific geographical locations (Deurenberg and Stobberingh, 2008; Chuang and Huang, 2013). Clones with multilocus ST59 and ST30 account for most of the CA-MRSA cases commonly reported in Asia (Chuang and Huang, 2013). The epidemiologic history of MRSA has been reshaped since the emergence of livestock-associated MRSA (LA-MRSA) in humans. Note that the predominant LA-MRSA lineage in Europe and North America is ST398, while in Asia, ST9 isolates are most prevalent (Chuang and Huang, 2015).

The reports of CA-MRSA have raised concerns about the prevalence and risk factors of MRSA in community populations. The prevalence of CA-MRSA varies substantially worldwide, ranging from less than 1% to more than 50% in different countries (Deurenberg and Stobberingh, 2008; Chuang and Huang, 2013). While some researchers have defined *S. aureus* (including MRSA) based on the clonal complex (CC) and resistance pattern, few have incorporated recent evidence suggesting that absence of the immune evasion cluster (IEC) genes may be a useful indicator of *S. aureus* livestock adaptation (Sung et al., 2008; Verkaik et al., 2011; Price et al., 2012; Rinsky et al., 2013). The IEC gene region at the 3′ end of βC-φs encodes up to four different immune evasion molecules [highly specific antiopsonic molecule (*scn*), chemotaxis and phagocyte activation (*chp*), inhibiting human α-defensins (*sak*), and staphylococcal enterotoxin (*sea*/*sep*)]; these mobile genetic elements move around in the population (van Wamel et al., 2006). The *scn* gene has been detected at a relatively high frequency among *S. aureus* isolates obtained from humans (90–100%) compared to those obtained from livestock (2–35%) (Sung et al., 2008; Verkaik et al., 2011; Price et al., 2012; Rinsky et al., 2013). A recent study in Germany indicated that IEC was absent from MRSA CC398 in pigs and pig farmers; however, IEC was found in MRSA CC398 from veterinary personnel (Cuny et al., 2015). Notably, there are few published studies of IEC genes among *S. aureus* isolates obtained from Asian populations, and it remains unclear whether there is a potential relation between the genetic lineages (ST types) and antibiotic resistance patterns and IEC genes.

In the present study, we aimed to examine the prevalence of carriage and antimicrobial susceptibility and the molecular characteristics (ST types and IEC genes) of MRSA and multidrug-resistant *S. aureus* (MDRSA) among healthy adults in Guangdong, China. We also investigated the potential relationship between the genetic lineages (ST types) of *S. aureus* isolates and antibiotic resistance patterns and IEC genes to reveal the potential risk of livestock-to-human transmission of livestock-associated *S. aureus* by non-occupational livestock contact.

## Materials and methods

### Ethics statement

This study was approved by the Ethics Committee of Guangdong Pharmaceutical University, and it was performed in accordance with the approved guidelines. All participants provided signed informed consent.

### Population and questionnaire

This cross-sectional study was conducted in Guangdong province, China, between November 2013 and November 2014. A multistage sample design was used to obtain a representative sample. First, four cities were randomly chosen from 21 cities in Guangdong province. Second, in each city, we selected two factories to achieve a sample size of 300 non-farm workers (i.e., workers from hardware factories or biscuit factories) with no occupational livestock contact. Finally, all workers in the selected venues were sampled to participate in this study. The following eligibility criteria were applied: (1) an age of 15 years or older, (2) an ability to speak and understand Chinese, (3) no current employment at a health care facility, (4) no acute infectious diseases in the last 7 days, and (5) no antibiotic use in the last 7 days. After obtaining informed consent, a face-to-face questionnaire was administered to collect demographic data and the influencing factors for *S. aureus* (including MRSA) carriage, such as sex, age (years), pet contact (e.g., dogs and cats) in homes in the last year, physical examination in a medical facility (including clinics, hospitals, community health station, and nursing homes) in the last month, antibiotic use in the last month and smoking (defined as “has smoked over 100 cigarettes in their lifetime and has smoked in the past month”). Note that none of the participants reported any occupational animal contact.

### Sample collection and culture methods

Two nasal swabs were taken from each participant (sterile cotton-wool swabs were used in both anterior nares), and the swabs were stored in 5 ml of enrichment broth containing 1% tryptone, 7.5% NaCl, 1% mannitol and 0.25% yeast extract at 4°C during transportation and incubated at 35 ± 1°C for 24 h. To isolate *S. aureus*, a loopful of the broth was plated on mannitol salt agar and incubated at 35 ± 1°C for 24–48 h. Per plate, one representative colony of each different suspected morphology was selected and purified on 5% sheep blood agar plates, and gram staining and catalase and coagulase tests were performed. *S. aureus* isolates were identified based on the above-mentioned positive tests.

### Antimicrobial susceptibility testing

All *S. aureus* isolates were tested for their susceptibility to 12 antimicrobials [i.e., penicillin (10 units), cefoxitin (30 μg), erythromycin (15 μg), clindamycin (2 μg), trimethoprim-sulfamethoxazole(25 μg), linezolid (30 μg), tetracycline (30 μg), rifampin (5 μg), chloramphenicol (30 μg), ciprofloxacin (5 μg), gentamicin (10 μg), and quinupristin-dalfopristin (15 μg)] using the Kirby-Bauer disk diffusion method, according to the Clinical and Laboratory Standards Institute guidelines 2013 (CLSI, 2013). *S. aureus* strain ATCC 25923 was used for the quality control. *S. aureus* isolates were classified as MDRSA if they were MRSA or non-susceptible to ≥3 classes of antibiotics (Magiorakos et al., 2012). *S. aureus* isolates resistant to cefoxitin were identified as suspect MRSA, according to CLSI (2013).

### Molecular characterization

All cefoxitin-resistant isolates were also tested through PCR for carriage of the *mecA* gene using the previously described primers (Zhang et al., 2004). The presence of IEC genes (*scn, chp, sak, sea*, and *sep*) was tested through PCR for all *S. aureus* isolates (van Wamel et al., 2006). Multilocus sequence typing (MLST) for all *S. aureus* is a nucleotide sequence-based approach for seven housekeeping genes. Alleles and sequence types were determined using the MLST database, and singletons or members of a clonal complex were determined using the eBURST algorithm (accessible at http://eburst.mlst.net).

### Statistical analysis

All data were entered in duplicate into an EpiData (version 3.1) database (the EpiData Association, Odense Denmark). The relationships between potential influencing factors and *S. aureus* and MRSA carriage were examined using Pearson's chi-squared test and multiple logistic regression models. We also conducted separate analyses to examine the phenotypic and molecular characteristics of *S. aureus* isolates between methicillin-sensitive *S. aureus* (MSSA) and MRSA isolates using Pearson's chi-squared test. A two-sided *p*-value for statistical significance was defined as *p* ≤ 0.05. All statistical analyses were performed using STATA version 14.0 (StataCorp LP, College Station, Texas, USA).

## Results

### Participant characteristics

The participant characteristics are provided in Table 1. A total of 1162 individuals were interviewed and participated in this survey. Approximately half of the participants were women (520/1162, 44.8%). The mean age (± standard deviation) was 36.7 ± 9.4 years, and the participant ages ranged from 15 to 60 years. Of the 1162 participants, 108 (9.3%) carried *S. aureus*, including 16 (1.4%) MRSA isolates. The overall prevalence of MDRSA carriage was 2.8% (32/1162).

**Table 1:** **Influencing factors of nasal carriage of ***S. aureus*** and MRSA among the 1162 study participants**.

**Influencing factors**	***n***	***S. aureus***			**MRSA**		
		***n*_1_(%)**	**OR(95%CI)**	***p*-value**	***n*_2_(%)**	**OR(95%CI)**	***p*-value**
**SEX**							
Female	520	63(12.1)	1.83(1.22–2.73)	0.003	6(0.9)	0.48(0.17–1.33)	0.205
Male	642	45(7.0)	1.00		10(1.9)	1.00	
**AGE (YEARS)**							
<35	439	52(11.8)	1.42(0.86–2.35)	0.053	9(2.1)	2.00(0.54–7.46)	0.306
35-45	433	31(7.2)	0.82(0.47–1.42)		4(0.9)	0.83(0.20–4.02)	
>45	290	25(8.6)	1.00		3(1.0)	1.00	
**PET CONTACT IN HOMES IN THE LAST YEAR**							
Yes	199	23(11.6)	1.35(0.83–2.20)	0.228	3(1.5)	1.12(0.32–4.00)	0.745
No	963	85(8.8)	1.00		13(1.3)	1.00	
**PHYSICAL EXAMINATION IN THE LAST MONTH**							
Yes	382	46(12.0)	1.59(1.06–2.37)	0.031	12(3.1)	6.29(2.02–19.64)	0.001
No	780	84(7.9)	1.00		4(0.5)	1.00	
**ANTIBIOTIC USE IN THE LAST MONTH**							
Yes	531	56(10.5)	0.76(0.51–1.13)	0.188	9(1.7)	0.65(0.24–1.76)	0.454
No	631	52(8.2)	1.00		7(1.1)	1.00	
**HOSPITALIZATION IN THE LAST YEAR**							
Yes	44	3(6.8)	0.71(0.21–2.31)	0.564	0(0.0)	–^a^	0.966
No	1118	105(9.4)	1.00		14(1.3)		
**SMOKING**							
Yes	532	30(5.6)	0.42(0.27–0.66)	<0.001	3(0.6)	0.32(0.09–1.15)	0.081
No	630	78(12.4)	1.00		11(1.8)		

*MRSA, methicillin-resistant S. aureus; n, number of participants; n_1_, number of participants carried with S. aureus; n_2_, number of participants carried with MRSA; –^a^, no odds ratio*.

### Influencing factors associated with *S. aureus* and MRSA carriage

The prevalence of *S. aureus* carriage was higher in females than in males (12.1% vs. 7.0%, *OR* = 1.83, 95% *CI* = 1.22–2.73). The prevalence of *S. aureus* carriage was lower in smokers than in no-smokers (5.6% vs. 12.4%; *OR* = 0.42, 95% *CI* = 0.27–0.66). Compared to individuals with no physical examinations, individuals with physical examinations experienced a significantly higher risk of *S. aureus* carriage (7.9% vs. 12.0%, *OR* = 1.59, 95% *CI* = 1.06–2.37) and MRSA carriage (0.5% vs. 3.1%; *OR* = 6.29, 95% *CI* = 2.02–19.64) (Table 1). In the multiple logistic regression model of sex, physical examination and smoking, there were significant differences in *S. aureus* carriage in terms of smoking status (adjusted *OR* = 0.45, 95% *CI* = 0.24–0.87) and physical examination (adjusted *OR* = 1.52, 95% *CI* = 1.01–2.31), but there was no significant difference in *S. aureus* carriage according to sex (adjusted *OR* = 1.06, 95% *CI* = 0.58–1.93).

### Antimicrobial susceptibility

The antimicrobial susceptibility among the 108 *S. aureus* isolates was based on CLSI (2013), with 92 (85.2%) classified as MSSA and 16 (14.8%) classified as MRSA (Table 2). Penicillin resistance was the most common phenotype observed in the MSSA (89.1%) and MRSA (100%) isolates. Compared with the MSSA isolates, the MRSA isolates had a higher risk of resistance to erythromycin (28.3% for MSSA vs. 62.5% for MRSA, *p* = 0.011), clindamycin (35.9% for MSSA vs. 68.8% for MRSA, *p* = 0.025), rifampin (3.3% for MSSA vs. 18.8% for MRSA, *p* = 0.041), and ciprofloxacin (3.3% for MSSA vs. 25.0% for MRSA, *p* = 0.009).

**Table 2:** **Phenotypic and molecular characteristics of MSSA and MRSA carried by the study participants in Guangdong, China**.

**Characteristics**	**Total *n* (%)**	**MSSA *n*_1_(%)**	**MRSA *n*_2_(%)**	***p*-value**
**PHENOTYPIC CHARACTERISTICS**				
Penicillin-resistant	98(90.7)	82(89.1)	16(100)	1.000
Erythromycin-resistant	36(33.3)	26(28.3)	10(62.5)	0.011
Clindamycin-resistant	44(40.7)	33(35.9)	11(68.8)	0.025
Tetracycline-resistant	35(32.4)	27(29.3)	8(50.0)	0.147
Trimethoprim-sulfamethoxazole-resistant	4(3.7)	3(3.3)	1(6.3)	0.479
Linezolid-resistant	3(2.8)	2(2.2)	1(6.3)	0.385
Rifampin-resistant	6(5.6)	3(3.3)	3(18.8)	0.041
Chloramphenicol-resistant	11(10.2)	9(9.8)	2(12.5)	0.666
Gentamicin-resistant	2(1.9)	1(1.1)	1(6.3)	0.276
Ciprofloxacin-resistant	7(6.5)	3(3.3)	4(25.0)	0.009
Quinupristin-dalfopristin-resistant	3(2.8)	1(1.1)	3(18.8)	0.057
**MOLECULAR CHARACTERISTICS**				
*scn-*positive	78(72.2)	67(72.8)	11(68.8)	0.766
*chp-*positive	36(37.5)	30(32.6)	6(37.5)	0.776
*sea-*positive	14(13.0)	12(13.0)	2(12.5)	1.000
*sak-*positive	70(64.8)	60(65.2)	10(62.5)	1.000
*sep*-positive	24(22.2)	21(22.8)	3(18.8)	1.000

*MSSA, methicillin-sensitive S. aureus; MRSA, methicillin-resistant S. aureus; n, number of S. aureus isolates; n_1_, number of methicillin-sensitive S. aureus; n_2_, number of methicillin-resistant S. aureus*.

The proportion of MDRSA among all *S. aureus* isolates was 29.6% (32/108), including 17.4% (16/92) among the MSSA isolates. Among the 16 multidrug-resistant MSSA isolates, the most common pattern of multiple resistance was non-susceptibility to erythromycin, clindamycin, and tetracycline (68.8%, 11/16). Among the 16 MRSA isolates, the most common pattern of multiple resistance was non-susceptibility to cefoxitin, erythromycin, and clindamycin (62.5%, 10/16; Table 3).

**Table 3:** **Antibiotic susceptibility patterns of multidrug-resistant ***S. aureus*** isolates carried by the study participants in Guangdong, China**.

**CC**	**MLST**	***scn***	***chp***	***sak***	***sea***	***sep***	**MRSA**	**Antibiotic resistance patterns**
CC1	ST1	+	−	+	+	−	Yes	FOX-TET-RIF
CC1	ST1	−	−	−	−	−	No	ERY-CLI-TET
CC5	ST5	−	−	+	+	−	Yes	FOX-TET
CC5	ST1863	+	−	+	−	−	No	ERY-CLI-TET
CC6	ST6	+	−	−	−	−	Yes	FOX-SXT-RIF
CC6	ST6	−	−	−	−	−	No	ERY-CLI-TET
CC6	ST6	+	−	+	+	+	No	ERY-CLI-SXT-RIF-QD
CC7	ST7	−	−	+	−	+	Yes	FOX
CC7	ST7	+	+	+	+	−	No	ERY-CLI-TET
CC7	ST7	+	−	+	−	−	No	ERY-CLI-TET
CC7	ST7	+	−	−	−	−	No	ERY-CLI-TET
CC7	ST7	−	−	−	−	−	Yes	FOX-ERY-CLI
CC7	ST7	−	−	−	−	−	Yes	FOX-ERY-CLI
CC7	ST7	−	−	−	−	−	Yes	FOX-CLI-TET-CIP
CC7	ST7	+	−	+	−	+	No	ERY-CLY-SXT-RIF-GEN
CC7	ST7	−	−	−	−	+	Yes	FOX-ERY-CLI-TET-CIP
CC7	ST7	−	−	−	−	−	Yes	FOX-ERY-CLI-TET-CIP
CC7	ST7	+	−	+	−	+	Yes	FOX-ERY-CLI-TET-CHL-QD
CC8	ST8	−	−	−	−	+	No	ERY-CLY-TET-CIP-CHL-LZD
CC10	ST10	+	+	+	−	−	Yes	FOX-ERY-CLI
CC15	ST15	+	+	−	−	+	No	ERY-CLI-TET
CC59	ST59	+	+	+	−	−	Yes	FOX
CC59	ST59	+	+	+	−	+	No	ERY-CLI-CHL
CC59	ST59	+	+	+	−	−	Yes	FOX-ERY-CLI
CC59	ST951	+	+	+	−	−	Yes	FOX-ERY-CLI
CC59	ST59	+	+	−	−	−	No	ERY-CLI-CHL
CC59	ST59	+	+	−	−	−	No	ERY-CLI-TET-CHL
CC59	ST59	+	+	−	−	−	No	ERY-CLI-TET-CHL
CC95	ST95	−	−	+	−	−	No	ERY−CLI−CHL
CC188	ST188	+	+	+	−	−	Yes	FOX-ERY-CLI-TET-RIF-CHL-CIP-LZD
CC398	ST398	−	−	−	−	−	No	ERY-CLI-TET-CHL-CIP
NT	NT	+	−	+	−	−	Yes	FOX-ERY-CLI-TET-CIP−GEN

*CC, clonal complex; ST, sequence type; +, positive; −, negative; FOX, cefoxitin; CLI, clindamycin; ERY, erythromycin; TET, tetracycline; CHL, chloramphenicol; SXT, trimethoprim-sulfamethoxazole; CIP, ciprofloxacin; RIF, rifampin; LZD, linezolid; GEN, gentamicin; QD, quinupristin-dalfopristin*.

### MLST typing and IEC genes

**Figure 1:**
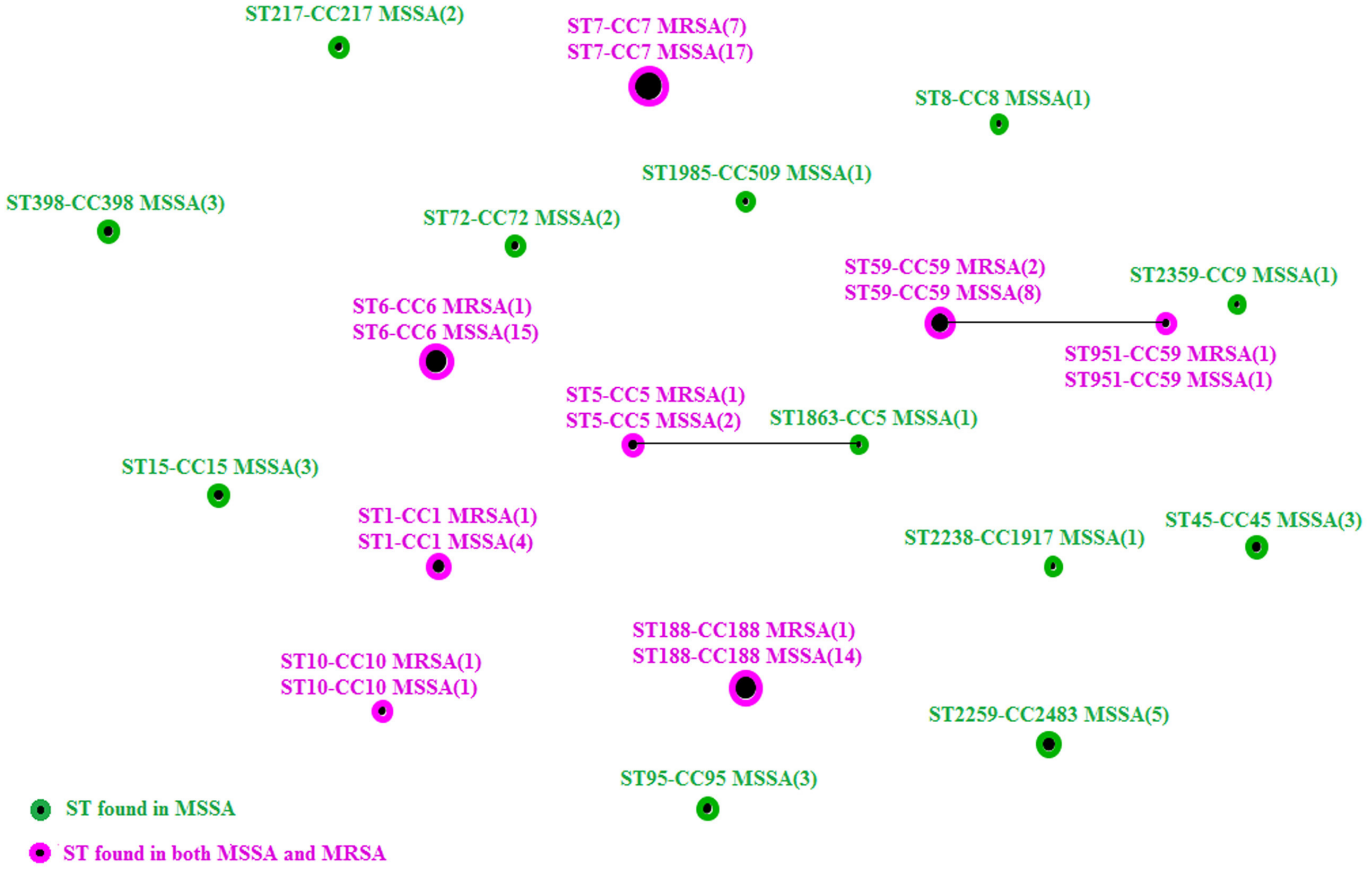
**A diagram produced using the eBURST algorithm with the stringent (default) group definition based on the MLST data from this study, comparing the relationship between MSSA and MRSA isolates**. (MSSA, methicillin-sensitive *S. aureus*; MRSA, methicillin-resistant *S. aureus*).

Twenty unique STs belonging to 18 CCs were identified from 108 *S. aureus* (Figure 1), except for 5 untypeable isolates. ST7 (24/108, 22.2%), ST6 (16/108, 14.8%), ST188 (15/108, 13.8%), and ST59 (10/108, 9.3%) were the most prevalent STs. Note that ST1, ST5, ST6, ST7, ST10, ST59, ST188, and ST951 were found in both MRSA and MSSA isolates.

In terms of the IEC genes, *scn* was present in 78 (72.2%) of the *S. aureus* isolates, *sak* was in 70 (64.8%), *chp* was present in 36 (37.5%), *sep* was present in 24 (22.2%), and *sea* was present in 14 (13.0%) (Table 2). Note that the proportions of IEC genes were similar in the MSSA and MRSA isolates (Table 2). Notably, the IEC(*sea* and *sep*)-negative MSSA CC9 (ST2359) was found in a pet owner, and the IEC(*scn, chp, sak, sea*, and *sep*)-negative tetracycline-resistant MDRSA ST398 and the IEC-negative (*scn, chp*, and *sep*) tetracycline-resistant MRSA ST5 were found in participants without pet contact (Table 3).

## Discussion

The results of the present study showed that 9.3% participants carried *S. aureus*, including 1.4% MRSA and 2.8% MDRSA, with the predominant multidrug-resistant pattern being non-susceptibility to erythromycin, clindamycin and tetracycline. The most common *S. aureus* genotypes were ST7, ST6, ST188, and ST59, with the predominant MRSA being ST7. Note that we found livestock-associated *S. aureus* isolates (IEC-negative CC9, IEC-negative tetracycline-resistant CC398, and IEC-negative tetracycline-resistant CC5) in participants with no occupational livestock contact, indicating a potential livestock-to-human transmission of livestock-associated *S. aureus* by non-occupational livestock contact.

*S. aureus* and MRSA nasal carriage in healthy adult population have been previously suggested to be approximately 10.7–37.1% and 0.2–8.6% (Gorwitz et al., 2008; Hamdan-Partida et al., 2010; Best et al., 2011; den Heijer et al., 2013; Wang et al., 2013; Yan et al., 2015), respectively, which is similar to our results (9.3% for *S. aureus* and 1.4% for MRSA). As reported in previous studies, the prevalences of *S. aureus* and MRSA were 10.7 and 1.9%, respectively, in Southeastern China (Wang et al., 2013) and 16.5 and 0.33%, respectively, in Northern China (Yan et al., 2015). Imparity results among healthy people in other countries have been recently reported. The carriage rates of *S. aureus* and MRSA were 30.4 and 1.2% in the United States (Gorwitz et al., 2008), 37.1 and 8.6% in Mexico (Hamdan-Partida et al., 2010), 18 and 0.2% in New Zealand (Best et al., 2011), and 21.6 and 0.3% in Europe (den Heijer et al., 2013), respectively. The prevalence of *S. aureus* in China is lower than that in American-European countries. There may be several reasons for this difference. In addition to geological and environmental factors, the prevalence of *S. aureus* can be influenced by study design (cross-sectional study or follow-up study), sampling sites (only anterior nares or multiple sites), screening and isolating methods for *S. aureus* (conventional biochemical methods or PCR test), and cultivation (enrichment or no enrichment).

Our study extends the previous knowledge in this area by identifying several predictors of *S. aureus* (including MRSA) carriage, which may help to inform clinical decision making when treating community-acquired infections. The finding that health care exposure (such as hospitalization, outpatient visit, surgery, and contact with healthcare workers) is significantly associated with MRSA carriage has been widely reported in the literature (Fritz et al., 2011; Rodriguez et al., 2014). Our study also indicated that physical examination was significantly associated with MRSA carriage. In addition, our study provides important evidence that smoking might be a protective factor against the nasal colonization of *S. aureus*, which aligns with previous studies (Wang et al., 2009; Olsen et al., 2012; Andersen et al., 2013). It might be that smoking creates a microenvironment in the nose that protects against the growth of *S. aureus*. A recent study conducted in northern Germany with a non-hospitalized adult population reported that males were more likely to carry *S. aureus* (Mehraj et al., 2014), indicating gender specific risk factors, which are not yet well understood but also align with observations from the USA, Norway and Denmark (Graham et al., 2006; Skråmm et al., 2011; Andersen et al., 2013). However, our study found no significant sex differences in *S. aureus* carriage after adjusting for smoking and physical examination, which is consistent with reports from Colombia (Rodriguez et al., 2014). Possible reasons for these findings include host genetics or human innate immune factors, hand-hygiene practice, smoking status, or vitamin D levels (Anderson et al., 2008; Johannessen et al., 2012; Olsen et al., 2012).

Infections caused by multidrug-resistant bacteria are associated with worse health outcomes and higher expenditures (Cardoso et al., 2012). However, few studies have examined the prevalence of MDRSA in healthy people. A recent study in North Carolina reported a 8.0% prevalence of MDRSA carriage among hog slaughter/processing plant workers compared with a 6.5% prevalence among household members, and the higher multidrug-resistant rate among workers may be associated with the use of multiple antimicrobials in hog feed (Neyra et al., 2014). In our study, the prevalence of multidrug-resistant *S. aureus* among health people was 2.8%, which was higher than a previous report of household members of livestock operation workers in America (0%) (Rinsky et al., 2013). Note that in our study, the most common pattern of multiple resistance among the participants with MSSA was non-susceptibility to erythromycin, clindamycin and tetracycline, and the most common pattern of multiple resistance among the participants with MRSA was non-susceptibility to cefoxitin, erythromycin and clindamycin, which aligned with a recent study of nine European countries (den Heijer et al., 2013). Erythromycin resistance mediates inducible macrolides, lincosamides and streptogramin B (MLSB), and phenotype erythromycin, together with the *erm* genes, encodes methylation of *23S rRNA* binding, which is commonly shared by clindamycin drug classes (Weisblum et al., 1971; Schwarz et al., 2002). In collaboration, erythromycin resistance and clindamycin resistance are mutually intensified, which causes MRDSA isolates to appear. Therefore, future studies must direct more attention to exploring the specific discrepancy of multiple resistance pattern and resistant genes among *S. aureus* species.

In our study, the most common *S. aureus* genotypes were ST7, ST6, ST188, and ST59, with ST7 as the predominant MRSA. Previously seldom noted in Chinese isolates, ST7 was the major clone among *S. aureus* isolates associated with skin and soft tissue infections (Yu et al., 2015). Notably, in a study of invasive community-acquired *S. aureus* infections in Chinese children, more than half of the ST7 isolates were associated with multi-site infections (Qiao et al., 2014). ST59 is a common genetic background among CA-MRSA isolates in Asian-Pacific populations, including children in Taiwan (Wang et al., 2004), outpatients in Japan (Yamaguchi et al., 2015), and skin and soft tissue infection patients in Australia (Coombs et al., 2010), but it has also been recently observed in healthy Spanish carriers (Argudin et al., 2013) and homeless youths in San Francisco (Pan et al., 2005).

It is well known that *S. aureus* CC398 and CC9 isolates colonize livestock and can spread to humans. Recent studies of *S. aureus* CC398 indicated that the best genetic marker for human-associated *S. aureus* (including MRSA) CC398 was positive for φ3 bacteriophage carrying IEC genes *chp* and *scn*, while the best genetic marker for livestock-associated *S. aureus* CC398 was positive for *tet*(M) (McCarthy et al., 2011, 2012). In addition, a follow-up study of American industrial hog operation workers reported that *scn*-negative *S. aureus* primarily belonged to CC398 and CC9 and 82% of *scn*-negative *S. aureus* isolates were tetracycline-resistant, but no *scn*-positive *S. aureus* demonstrated tetracycline resistance (Nadimpalli et al., 2015). In our study, the livestock-associated IEC-negative tetracycline-susceptible MSSA CC9 (ST2359) was found in a participant with pet contact, and the livestock-associated IEC-negative tetracycline-resistant MDRSA ST398 was found in a participant without pet contact. Therefore, our findings support growing concern about the potential livestock-to-human transmission of livestock-associated *S. aureus* by non-occupational livestock contact.

Several studies have indicated that *S. aureus* ST5 isolates are relatively common in the swine industry in America and European countries (Frana et al., 2013; Hau et al., 2015; Linhares et al., 2015). In contrast to ST398 and ST9, the presence of ST5 in swine has raised additional public health concerns because ST5 is among the most prevalent lineages causing clinical infections in humans (Hau et al., 2015). The prominence of ST5 in clinical disease is believed to result from the acquisition of bacteriophages containing virulence and IEC genes. Previous studies have revealed that none of the swine isolates contained IEC genes (*scn* and *sak*), while these genes were present in 90% of the isolates from humans with no swine contact (Hau et al., 2015). Additionally, MRSA ST5 isolates of chicken origin were tetracycline resistant and had no IEC genes (*sea, sak, scn*, and *chp*) (Monecke et al., 2013). Similarly, the livestock-associated IEC-negative (*scn, chp*, and *sep*) tetracycline-resistant MRSA ST5 was found in a participant with no pet contact in our study, and some possible modes of exposure, including contact with air or waste releases from livestock farms, contact with contaminated meats and animals, and person-to-person contact, could not be ruled out.

This study contributed to the literature by exploring the potential relationship between genetic lineages (ST types) of *S. aureus* isolates and antibiotic resistance patterns and IEC genes. However, some limitations should be considered when interpreting our results. First, this study was conducted at only one time point (using a cross-sectional design), thus, we could not understand whether the carriage of MRSA among healthy people was transient or persistent. At the same time, we can only describe associations between influencing factors and MRSA carriage, not a causal conclusion. The results from this study must be confirmed and improved in a longitudinal study. Second, only the anterior nares were used as a sampling site, which may underestimate the true prevalence of *S. aureus* and MRSA. However, these measures have been widely used in previous similar studies in other countries, and the results were consistent with previous studies, as noted above. In addition, in this large population study, to simplify the collection of microbial samples and to compare the results with other similar studies, we chose to limit the data collection to nasal swabs. Third, a novel *mecA* homolog denominated *mecC* has been found in *S. aureus* lineages typically associated with cattle (i.e., CC130, CC1943, and C425). Although screening MRSA using cefoxitin allows the identification of *mecC*-MRSA, further studies are need to identify the *mecC* gene among *S. aureus* being phenotypic MRSA but negative for *mecA*.

In conclusion, this study provides insight into the phenotypic and genotypic determinants of *S. aureus* (including MRSA and MDRSA) isolates from healthy adults, and finds a potential relationship between *S. aureus* CCs and IEC genes and antibiotic resistance patterns in defining livestock-associated *S. aureus* in humans. The livestock-associated *S. aureus* isolates were found in participants with no occupational livestock contact; therefore, these findings support growing concern about the potential livestock-to-human transmission of livestock-associated *S. aureus* by non-occupational livestock contact.

## Author contributions

YF and XW carried out the samplings, the molecular genetic studies and drafted the manuscript. YF, XW, and LL participated to the samplings and performed the experiments. ZY participated in the design of the study. XY and SC coordinated the study and helped to draft the manuscript. All authors read and approved the final manuscript.

## Funding

This work was supported by the National Natural Science Foundation of China (No. 81602901) and the Innovation and Strong School Project of Guangdong Pharmaceutical University (No. 2014KQNCX138). The funders played no role in the study design or data collection, analysis, and interpretation.

### Conflict of interest statement

The authors declare that the research was conducted in the absence of any commercial or financial relationships that could be construed as a potential conflict of interest.
